# Extract from *Armoracia rusticana* and Its Flavonoid Components Protect Human Lymphocytes against Oxidative Damage Induced by Hydrogen Peroxide

**DOI:** 10.3390/molecules19033160

**Published:** 2014-03-14

**Authors:** Michala Gafrikova, Eliska Galova, Andrea Sevcovicova, Petronela Imreova, Pavel Mucaji, Eva Miadokova

**Affiliations:** 1 Department of Genetics, Faculty of Natural Sciences, Comenius University, Mlynská dolina, Bratislava 842 15, Slovakia; 2 Department of Pharmacognosy and Botany, Faculty of Pharmacy, Comenius University, Odbojárov 10, Bratislava 832 32, Slovakia

**Keywords:** *Armoracia rusticana*, kaempferol, quercetin, oxidative damage, hydrogen peroxide, comet assay

## Abstract

DNA damage prevention is an important mechanism involved in cancer prevention by dietary compounds. *Armoracia rusticana* is cultivated mainly for its roots that are used in the human diet as a pungent spice. The roots represent rich sources of biologically active phytocompounds, which are beneficial for humans. In this study we investigated the modulation of H_2_O_2_ genotoxicity using the *A. rusticana* root aqueous extract (AE) and two flavonoids (kaempferol or quercetin). Human lymphocytes pre-treated with AE, kaempferol and quercetin were challenged with H_2_O_2_ and the DNA damage was assessed by the comet assay. At first we assessed a non-genotoxic concentration of AE and flavonoids, respectively. In lymphocytes challenged with H_2_O_2_ we proved that the 0.0025 mg·mL^−1^ concentration of AE protected human DNA. It significantly reduced H_2_O_2_-induced oxidative damage (from 78% to 35.75%). Similarly, a non-genotoxic concentration of kaempferol (5 μg·mL^−1^) significantly diminished oxidative DNA damage (from 83.3% to 19.4%), and the same concentration of quercetin also reduced the genotoxic effect of H_2_O_2_ (from 83.3% to 16.2%). We conclude that AE*,* kaempferol and quercetin probably act as antimutagens. The molecular mechanisms underlying their antimutagenic activity might be explained by their antioxidant properties.

## 1. Introduction

The human body and cells are daily exposed to negative effects of many DNA damaging agents from food or the environment, such as ultraviolet or ionizing radiation, viruses, alkylating or oxidative agents. These agents can cause DNA damage (single- or double-strand breaks representing primary DNA lesions leading to a fixation of mutations through misrepair or misreplication). They also have an influence on the functions of lipids, and proteins and are able to destroy the cell membrane or the whole cell compartment.

One of such agents is hydrogen peroxide. It is normally produced in cells as a by-product of oxidative metabolism. Under normal conditions it is reduced to water by catalase, glutathione peroxidases and peroxiredoxins [1]. When reduction mechanisms are not sufficient, hydrogen peroxide can react with transition metals (iron, copper) and via the Fenton reaction they together produce highly reactive hydroxyl radical which attacks DNA at the sugar residue of the DNA backbone, and this leads to DNA single-strand breaks. They also transform purines and pyrimidines to their corresponding hydroxyl derivatives, such as 8-hydroxyguanine [2]. Reactive oxygen species (e.g., hydrogen peroxide, superoxide radical, *etc.*) can cause oxidative damage that negatively influences the function of proteins, induces mutations in nucleic acid and causes lipid peroxidation [3].

It is very important to find agents that are able to protect the human body and cells and decrease the DNA damage induced by genotoxic agents. Our attention was focused primarily on plant extracts and their active components. The natural extracts and their components can be used to produce natural medicines that are safe for a human body. Moreover, they are normally safer than synthetic drugs due to their minimum side effects. Other advantages of natural medicines are their availability, biodegradability and greater acceptance amongst end users. They are safe not only for mankind but for the environment too [4].

Plants are very important for human everyday life. People use them as a part of a normal diet, in cosmetics and pharmaceutical products. Plants are also used for the production of drinks (tea, coffee, wine). Detailed knowledge about the biological effects of plants and their components on human organisms is very important due to their immune system’s stimulation ability as well as their disease prevention potential.

The horseradish, *Armoracia rusticana* (P. Gaertn., B. Mey. & Scherb.), belongs to the genus *Armoracia* of the family *Cruciferae*. It is a perennial crop which is cultivated mainly in Europe and Asia because its roots are used in the human diet as a pungent spice. The roots are also rich sources of biological compounds beneficial for humans [5,6]. The interest in the investigation of bioactive components, especially phenolic compounds, from natural sources has greatly increased in recent years [7]. Besides phenolic compounds, there are also enzymes of great interest. Peroxidase, (EC 1.11.1.7), produced by horseradish, is a heme-containing enzyme utilizing hydrogen peroxide in the oxidation of many organic and inorganic compounds [8]. Myrosinase (β-thioglucoside glucohydrolase, EC 3.2.3.147) is also one of many components of *Armoracia rusticana* roots [9].

Glucosinolates are present in the roots of *A. rusticana*. Sinigrin, glucobrassicin, neoglucobrassicin and gluconasturin were detected in major quantities [10]. The roots also contain ascorbic acid (vitamin C) that is very important for humans who are not able to synthesize it. Ascorbic acid is a very strong antioxidant and it also plays a role in collagen synthesis [11,12,13]. *Armoracia rusticana* contains a small amount of flavonoids – kaempferol and quercetin [14,15,16,17,18]. 

The aim of this study was the genotoxicological research of the aqueous plant extract from *Armoracia rusticana* and two flavonoids, kaempferol and quercetin, these being the main flavonoid components of this extract.

Flavonoids represent a group of over 8,000 naturally occurring polyphenolic compounds that are ubiquitous in the plant kingdom. They are present for example in onions, kale, broccoli, apples, cherries, tea, parsley, grapes or soybeans. Depending on the various combinations of hydroxyl and methoxyl group substituents on the basic flavonoid skeleton they can be classified as follows: flavonols, flavones, chalcones, flavanones, anthocyanidins and isoflavonoids. These natural compounds are the subject of extensive scientific and clinical research nowadays [19,20].

**Figure 1:**
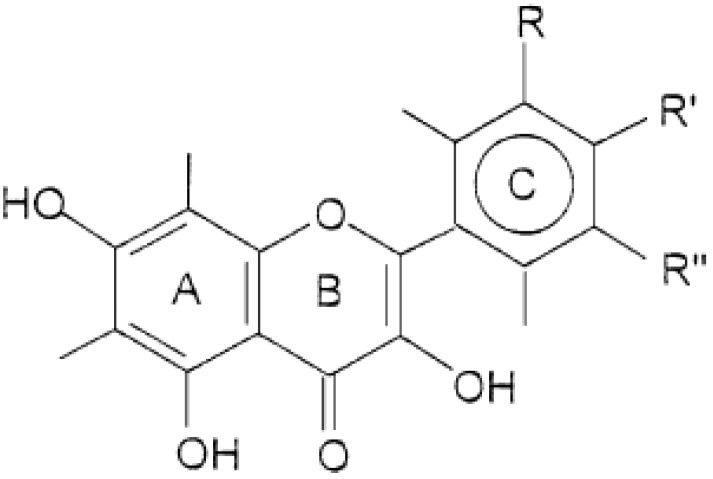
Primary structure of flavonoles; R, R', R'' - substituents.

The flavonoids kaempferol and quercetin investigated in this research, belong to the flavonol subclass of flavonoids [21]. Flavonoles and 2-phenyl-3-hydroxychromanes have similar primary structures (Figure 1) [22]. Kaempferol has a hydroxyl group at the R' position and the R and R'' positions are free. Quercetin has two hydroxyl groups at the R' and R'' positions and the position R is free [23].

Kaempferol is a yellow compound with a low molecular weight (MV: 286.2 g.mol^−1^). It is one of many components in foodderived from plants and also in plants that are used in traditional medicine (e.g., *Ginkgo biloba*) [24]. Many researchers have proven that kaempferol has a positive biological effect on a human body and health. Kaempferol has the ability to induce apoptosis in glioblastoma cells under the oxidative stress conditions. It supports the production of proapoptotic molecules – active caspase 3 and poly(ADP-ribose) polymerase (PARP) protein. On the other hand kaempferol decreases the expression of the antiapoptotic protein Bcl-2 and also the mitochondrial membrane potential which leads to apoptosis. Treatment of cells with kaempferol minimizes the expression of superoxide dismutase and thioredoxin that helps maintain the redox balance [25].

Quercetin, 3,3',4',5,7-pentahydroxyflavone, is ubiquitous in plants and it is the major bioflavonoid in the human diet [26]. This flavonoid has a positive effect on the human organism because of its antioxidant properties. It can also decrease the oxidative damage caused by ethanol in mice [27]. Quercetin induces apoptosis in HeLa cells because it inhibits the heat shock proteins Hsp27 and Hsp72 [28]. Both quercetin and kaempferol exhibit protective effects on human lymphocytes and sperm against two dietary mutagens: 3-amino-1-methyl-5*H*-pyrido(4,3-b)indole (Trp-P-2) and 2-amino-3-methylimidazo(4,5-f)quinoline (IQ) [29]. 

The studies undertaken with the aim to present the bioprotective (antimutagenic, antioxidant *etc.*) power of a plant extract on the basis of its flavonoid components have, in most cases, failed due to antagonistic interactions between flavonoids. Quercetin and kaempferol are exceptional because their synergistic antioxidant activity was proven [30]*.* We anticipated that such an activity could also contribute to the final antigenotoxic activity of the extract. To study this, we searched for non-genotoxic concentrations of the extract and flavonoids. These concentrations were subsequently used to investigate the ability of the extract and flavonoids to modulate the DNA damage induced by hydrogen peroxide in freshly isolated human lymphocytes.

## 2. Results and Discussion

### 2.1. Non-Genotoxic Concentration of A. rusticana Extract and Flavonoids

In our study, we wanted to test whether the pre-incubation of lymphocytes with the *A. rusticana* extract or flavonoids can decrease the hydrogen peroxide-induced DNA damage. We used the hydrogen peroxide challenge assay which is a method used widely to detect the antigenotoxic potential of various plant extracts. It enables one to assess the capacity of plant extracts and their components to protect DNA against DNA oxidation in human cells [31]. First we tried to find non-genotoxic concentrations of the extract and flavonoids.

**Figure 2:**
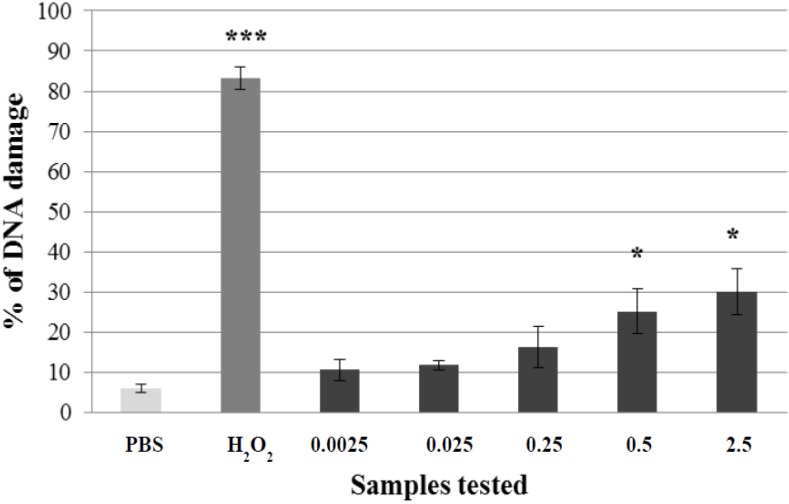
Potential genotoxic activity of different concentrations of *A. rusticana* extract (AE) tested on lymphocytes using the comet assay. Legend for the x axis: PBS = negative control, H_2_O_2_ = positive control, 0.0025–2.5 = samples treated with AE (concentration in mg·mL^−1^). All experiments were performed at least three times. Mean values ± SD. * = comparison with negative control. * *p* < 0.05; ** *p* < 0.01; *** *p* < 0.001.

We evaluated a wide range of concentrations of the extract and flavonoids using the comet assay. For *A. rusticana* extract, a range of five concentrations from 0.0025 to 2.5 mg·mL^−1^ was tested. Three concentrations of the aqueous extract from *A. rusticana* (0.0025 mg·mL^−1^; 0.025 mg·mL^−1^; 0.25 mg·mL^−1^) did not exhibit any genotoxic activity and could be considered as non-genotoxic. Two higher concentrations of the extract from *A. rusticana* (0.5 mg·mL^−1^; 2.5 mg·mL^−1^) showed a low genotoxic activity (*p* < 0.05) (Figure 2). For further experiments we chose the concentration 0.0025 mg·mL^−1^.

While searching for non-genotoxic concentrations of flavonoids, we evaluated a range of eleven concentrations from 5 to 1,500 μg·mL^−1^. At first we tested a range from 250 to 1,500 μg·mL^−1^. We did not find a non-genotoxic concentration because all the concentrations exhibited a low or moderate DNA damage (from *p* < 0.05 to *p* < 0.001) (data not shown). 

Higher concentrations of kaempferol (from 500 to 1500 μg·mL^−1^) showed some DNA damage comparable to the same concentrations of quercetin (from *p* < 0.05 to *p* < 0.001). Our results are in agreement with the results obtained by other authors who used these flavonoids *in vitro* and *in vivo* [32]. Therefore, we applied lower concentrations of flavonoids in the range from 5 to 100 μg·mL^−1^.

**Figure 3:**
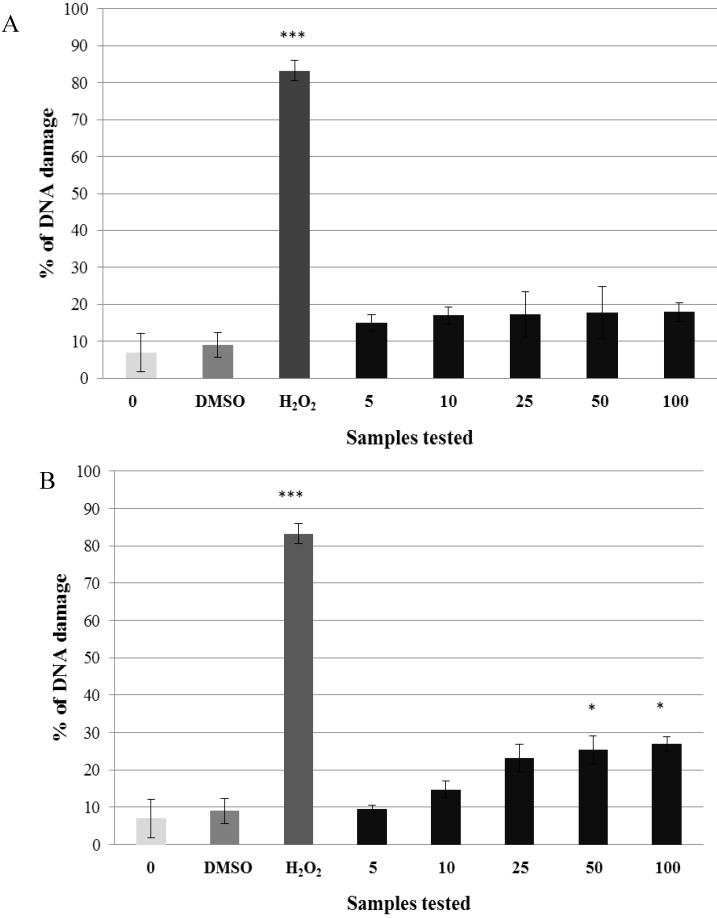
Potential genotoxic activity of different concentrations of kaempferol (**A**) and quercetin (**B**) tested on lymphocytes using the comet assay. Legend for the x axis: 0 = PBS, DMSO = solvent, H_2_O_2_ = positive control, 5–100 = samples treated with flavonoid (concentration in μg·mL^−1^). All experiments were performed at least three times. Mean values ± SD. * = comparison with negative control. * *p* < 0.05; ** *p* < 0.01; *** *p* < 0.001.

Kaempferol concentrations in the range from 5 to 100 μg·mL^−1^ exerted a non-genotoxic effect (Figure 3A). The three lowest concentrations of quercetin (from 5 to 25 μg·mL^−1^) were not genotoxic either. Two higher concentrations (50 μg·mL^−1^; 100 μg·mL^−1^) showed a low genotoxic effect (Figure 3B). Based on these results we chose a non-genotoxic concentration 5 μg·mL^−1^ of kaempferol and quercetin for further experiments.

### 2.2. Pre-Incubation of Lymphocytes with Non-Genotoxic Concentrations of A. rusticana Extract and Flavonoids Decreased the DNA Damage Induced by Hydrogen Peroxide

**Figure 4:**
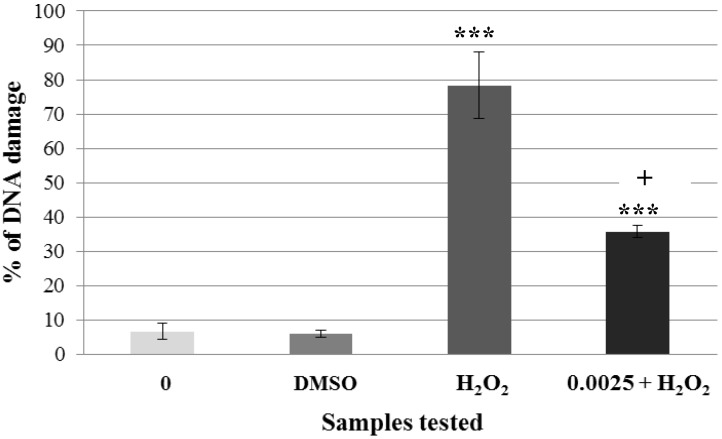
Pre-incubation of lymphocytes with a non-genotoxic concentration of the extract from *A. rusticana* (AE). Legend for the x axis: 0 = negative control (PBS), H_2_O_2_ = positive control; 0.0025 = sample treated with AE (concentration in mg·mL^−1^), 0.0025 + H_2_O_2_ = sample pre-treated with AE (concentration in mg·mL^−1^) and treated with H_2_O_2_. All experiments were performed at least 3 times. Mean values ± SD. * = comparison with negative control. * *p* < 0.05; ** *p* < 0.01; *** *p* < 0.001. ^+^ = comparison with positive control. ^+^ *p* < 0.05; ^++^ *p* < 0.01; ^+++^ *p* < 0.001.

After the selection of non-genotoxic concentrations of *A. rusticana* extract and flavonoids, we tested whether the pre-treatment of human lymphocytes challenged with hydrogen peroxide has the ability to protect human DNA. Firstly, non-genotoxic concentration of the aqueous extract from *A. rusticana* (0.0025 mg·mL^−1^) was tested (Figure 4).

Lymphocytes without treatment (DNA damage was 6.6%) were used as the negative control. Lymphocytes treated only with hydrogen peroxide were used as the positive control (DNA damage was 78%). Lymphocytes incubated with non-genotoxic concentration of *A. rusticana* extract caused only 10.6% of DNA damage. After the pre-incubation of lymphocytes with non-genotoxic concentration prior to hydrogen peroxide exposure, the DNA damage reached only 35.75% (Figure 4). This result proves that non-genotoxic concentration (0.0025 mg·mL^−1^) of *A. rusticana* extract has the ability to decrease DNA damage induced by hydrogen peroxide. We detected a reduction from 78% to 35.75% compared to the positive control (lymphocytes treated with hydrogen peroxide only). We obtained very similar results to the ones obtained after the pre-incubation of lymphocytes and HEK 293 cells with the extract from *Gentiana asclepiadea* [33].

**Figure 5:**
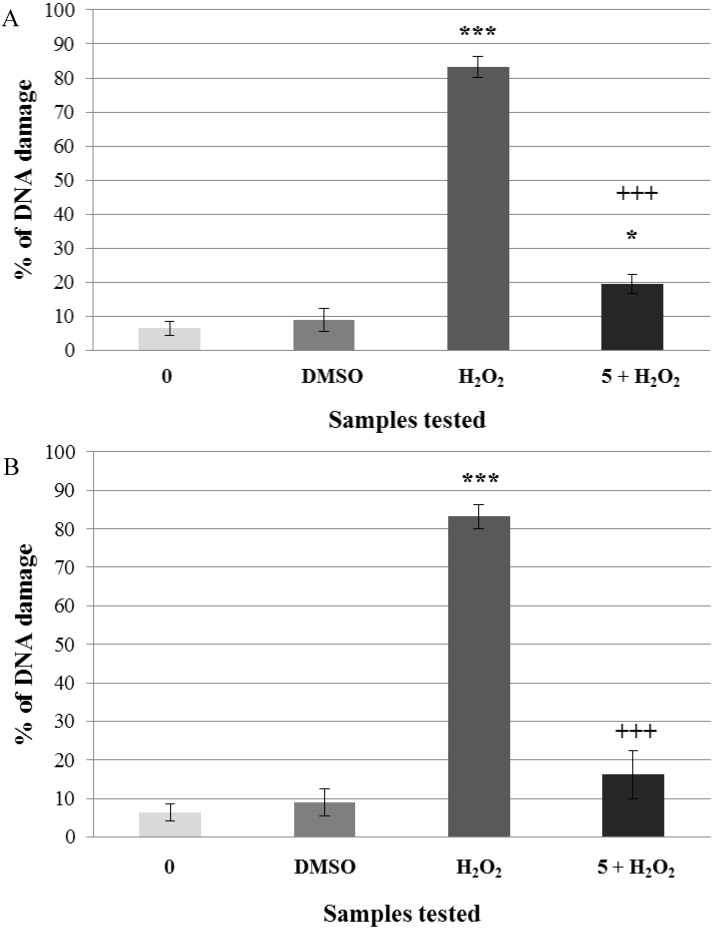
Pre-incubation of lymphocytes with a non-genotoxic concentration of flavonoids (A = kaempferol, B = quercetin). Legend for the x axis: 0 = negative control (PBS); DMSO = solvent; H_2_O_2_ = positive control; 5 = sample treated with flavonoid (concentration in μg·mL^−1^), 5+H_2_O_2_ = sample pre-treated with kaempferol (**A**) or quercetin (**B**) (concentrations in μg·mL^−1^) and treated with H_2_O_2_. All experiments were performed at least three times. Mean values ± SD. * = comparison with negative control. * *p* < 0.05; ** *p* < 0.01; *** *p* < 0.001. ^+^ = comparison with positive control. ^+^ *p* < 0.05; ^++^ *p* < 0.01; ^+++^ *p* < 0.001.

We finally tested the non-genotoxic concentrations of flavonoids—5 μg·mL^−1^. For the negative control, we used lymphocytes incubated in PBS (DNA damage was 6.4%). Lymphocytes treated with hydrogen peroxide were used as the positive control (DNA damage was 83.25%). Non-genotoxic concentration of kaempferol induced higher DNA damage (15%) than the quercetin one (9.6%). After the pre-incubation with kaempferol prior to hydrogen peroxide treatment, we found out a decrease of DNA damage from 83.25% to 19.4%. After the pre-incubation with quercetin prior to hydrogen peroxide exposure, the percentage of DNA damage significantly decreased from 83.25% to 16.2% (Figure 5A,B). Both flavonoids significantly reduced hydrogen peroxide-induced DNA damage.

In the present study, we demonstrated that the aqueous extract from *A. rusticana* and the flavonoids kaempferol and quercetin prevented the induction of single-strand DNA breaks. We propose that the extract from *A. rusticana* and flavonoids might be considered as desmutagens. Desmutagens are defined as agents being able to suppress mutations by decreasing levels of DNA lesions (including single-strand breaks as primary DNA lesions) through various mechanisms. They can suppress mutations by decreasing levels of DNA lesions via their antioxidant (scavenging, transient metals chelating) properties, or by preventing or decreasing the conversion of pro-mutagens to ultimate mutagens. They have also the ability to degrade/detoxificate mutagens or induce enzymes that can detoxificate mutagen prior to reaching DNA [34,35].

In our preliminary experiments scavenging/antioxidant activities of the extract and both flavonoids were assessed (unpublished data), so that we could hypothesize that the underlying mechanism of H_2_O_2_genotoxicity reduction might be a result of the antioxidant activity of *A. rusticana* and the tested flavonoids. It is necessary to realize that the combinations of various flavonoids usually promote antagonistic effects. Kaempferol and quercetin are unique in their synergistic antioxidant activity [30]. We propose that this synergistic antioxidant activity can effectively contribute to the overall antigenotoxic effect of *A. rusticana* extract. We obtained similar results when comparing the effect of lymphocytes pre-incubation with methanolic extracts from *A. rusticana* and *Gentiana asclepiadea*. All of them had the ability to modulate DNA damage induced by hydrogen peroxide in the case of pre-incubation and acted as desmutagens [36]. We also came to the conclusion that methanolic extracts from *A. rusticana*, *G. asclepiadea* had a lower modulating effect than the aqueous extract from *A. rusticana* presented in this study. Differences between these results might be due to the fact that different extracts obtained from the same plant (aqueous or methanolic) may differ not only in the quantity of their components, but even in their chemical composition. As the antimutagenic activity of natural compounds often correlates with the antioxidant activities [37], we could consider that the molecular mechanisms underlying their antimutagenic effect might be explained by their antioxidant potential (attributed to the free radicals capture).

Our results correlate with the study in which kaempferol or quercetin pre-treated HepG2 cells were exposed to a genotoxic agent – benzo[a]pyrene [38]. The results from this study demonstrated that the pre-incubation with flavonoids decreased DNA damage. Their unique structure and varied pharmacological activities may bring new possibilities for a discovery of drugs with a new mechanism of action [39].

## 3. Experimental

### 3.1. Preparation of Armoracia rusticana Plant Extract

The air-dried plant material (roots) weighing about 70 g was cut into small pieces and then extracted to 150 mL of water at 65 °C. This procedure was repeated five times. The hot solution of the extract was then filtered and concentrated using a vacuum evaporator. The final aqueous extract from the roots of *A. rusticana* was kept in the dark at 4 °C until tested, and then diluted in 1× PBS.

### 3.2. Flavonoids Preparation

Both flavonoids, kaempferol and quercetin, were purchased from Sigma-Aldrich (Bratislava, Slovakia). They were dissolved in DMSO solution (1%) and kept in the dark at 4 °C until tested.

### 3.3. Lymphocytes

Lymphocytes were obtained from peripheral blood using the *finger prick* method just prior to use. Blood (40–50 μL) was taken and added to phosphate buffer solution (1 mL, 1× PBS, pH 7.5), mixed and left on ice up to 30 min. Then we underlayed it with Histopaque 1077 (Sigma, 100 μL) and spinned at 180 ×*g* for 5 min at 4 °C. Lymphocytes (100 μL) were retrieved from just above the boundary between the phosphate buffer and Histopaque, pipetted into new Eppendorf tubes with 1 mL of PBS and spinned again at 180 ×*g* for 5 min at 4 °C. Supernatant was removed and the lymphocytes were used for the subsequent analyses.

### 3.4. Comet Assay

The comet assay was based on the method of Collins *et al*. [40]. Briefly, prior to the assay we prepared various concentrations of the aqueous extract from *A. rusticana* roots and of flavonoids. Lymphocytes placed on cold-resistant microscope slides were incubated with various concentrations of *A. rusticana* extract or flavonoids and covered for 30 min in wet room at 37 °C. Two samples were used as negative controls (1× PBS; 1% DMSO). Another sample was immediately treated with hydrogen peroxide (35 μM) for 5 min at 4 °C, and served as the positive control. All samples were placed in a lysis solution (pH 10.0) for 1 h at 4 °C to remove cellular membrane and cytoplasm while leaving nucleoids. After lysis, the samples were placed to electrophoretic tank with alkaline solution (pH 13.0) for 20 min at 4 °C for DNA unwinding. After unwinding, the electrophoresis was carried out under the following conditions: 30 min, 25 V, 260–320 mA at 4 °C. The samples were neutralised for 7 min in PBS (pH 7.5) and then 7 min in deionized water at 4 °C. Nucleoids were analysed by at 100× magnification using an Olympus BX51 fluorescence microscope equipped with a U-MNU2 filter and captured by the Olympus U-CMAD3 Color View Soft Imaging System. Images were analysed with the image analysis software CometScore™ (TriTec Corporation, San Diego, CA, USA). Comets were classified into five categories: 0 representing undamaged cells (comets with no or barely detectable tails) and 1–4 representing increasing relative tails intensities. Summing the scores (0–4) of 100 comets gives an overall score between 0 and 400 arbitrary units. The percentage of the DNA damage was subsequently evaluated with the image analysis software CometScoreTM (TriTec Corporation).

### 3.5. H_2_O_2_ Challenge Assay

Isolated human lymphocytes were pre-incubated in the wet room at 37 °C in the dark with non-genotoxic concentrations of the extract from *A. rusticana* or flavonoids. After the pre-incubation lymphocytes were washed in the phosphate buffer (1× PBS, pH 7.5), incubated in H_2_O_2_ (35 μM) for 5 min at 4 °C and washed again in the phosphate buffer. Cells used for the positive control were immediately treated with in H_2_O_2_ for 5 min at 4 °C. Afterwards, the lymphocytes were submitted to the comet assay.

### 3.6. Statistical Analysis

The results represent the mean of three experiments ± standard deviation. The significance of differences between means was evaluated by the Student’s t-test: * *p* < 0.05; ** *p* < 0.01; *** *p* < 0.001.

## 4. Conclusions

Our study has documented the great potential of the aqueous plant extract from *A. rusticana* and its main flavonoids, kaempferol and quercetin, to protect DNA from damage induced on human lymphocytes by the oxidative agent hydrogen peroxide. DNA damage prevention is an important mechanism in cancer chemoprevention by dietary compounds. We proved that naturally occurring plants and their components can prevent against negative impacts on human lymphocytes and these results can be potentially useful for pharmacology and medicine.
